# Supplementary figures and images for: LAP: Liability Antibody Profiler by sequence & structural mapping of natural and therapeutic antibodies
Source: PLoS Comput Biol. 2024 Mar 5;20(3):e1011881. doi: 10.1371/journal.pcbi.1011881 (PMC10957075; doi:10.1371/journal.pcbi.1011881)

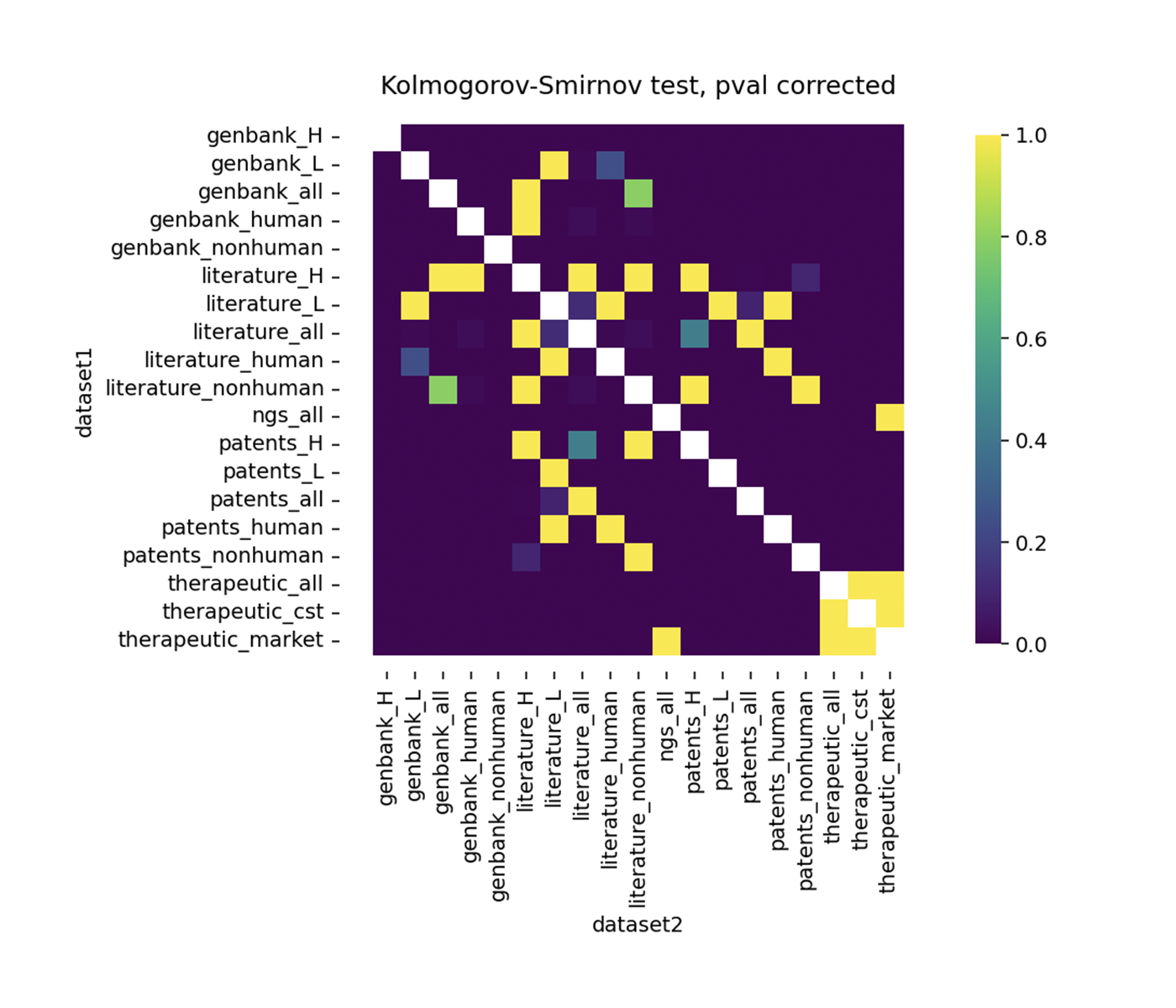

Supplement: S1 Fig — Chi2 test was applied to test the difference in number of liabilities per sequence between any two datasets. Because there were multiple tests, Bonferroni correction was applied. Abbreviations after the underscore mean respectively: “H”—heavy chain, “L”- light chain, “all”—all sequences,“human”—only human antibody sequences, “nonhuman”—only non humanantibody sequences, “cst”—clinical stage therapeutics, “market” -therapeutics on the market. (TIF) [file pcbi.1011881.s001.tif]

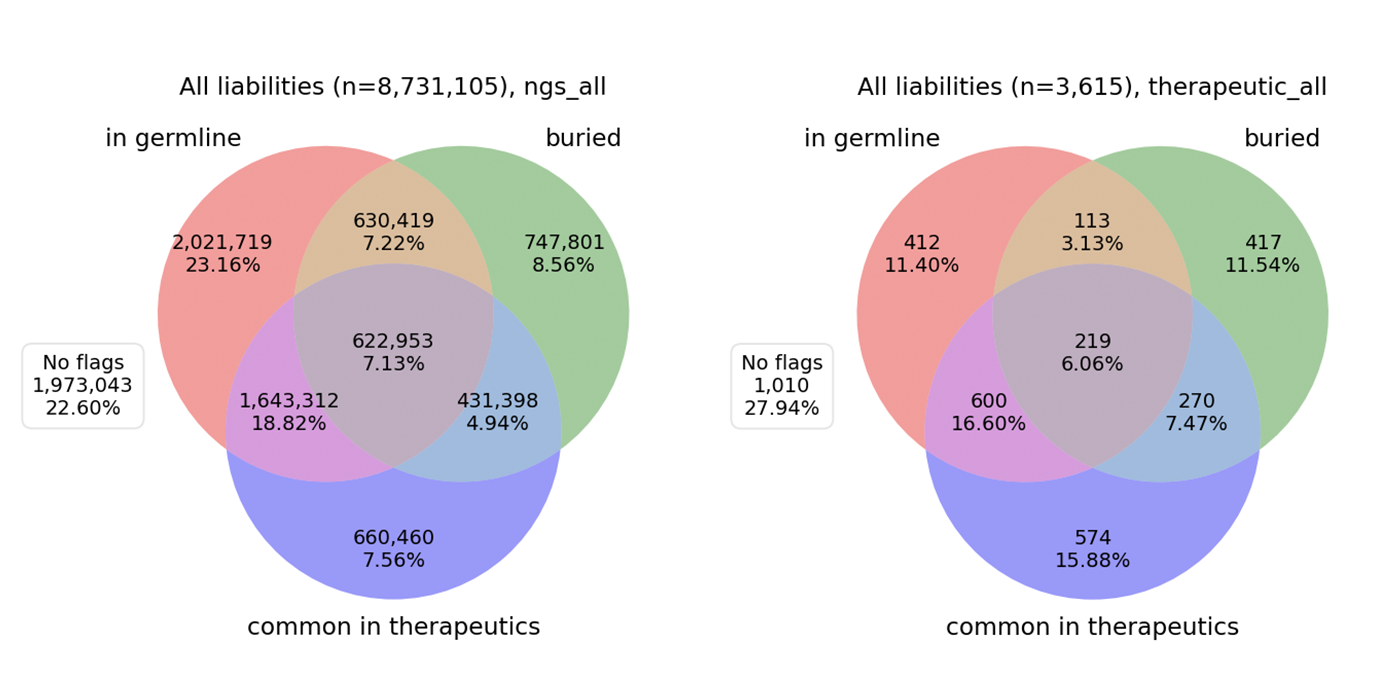

Supplement: S2 Fig — (TIF) [file pcbi.1011881.s002.tif]
