# Supplementary material for: LAP: Liability Antibody Profiler by sequence & structural mapping of natural and therapeutic antibodies
Source: PLoS Comput Biol. 2024 Mar 5;20(3):e1011881. doi: 10.1371/journal.pcbi.1011881 (PMC10957075; doi:10.1371/journal.pcbi.1011881)
Supplement: Attachment — Submitted filename: LAP Reviewer Response.docx [file pcbi.1011881.s003.docx]

We would like to thank the editors and reviewers for their input that strengthens our work.We hope that our revised manuscript and corrections will satisfy your requirements. Responses are given below in canonical point-by point fashion with modified text highlighted in one submitted version.

Reviewer 1.

**1. It isn't clear how the authors arrived at the motifs defined in Table 1. Does this information come from conversations and/or unpublished work or are there published papers that the researchers can cite to clarify this? The value of this method is dependent on the validity of these motifs, so it would be useful to understand where they come from.**

We apologize for not making it clearer as it is quite a crucial point. Since there is no ‘set’ set of liabilities we did our best at systematizing these employing 1) scientific publications 2) running a survey across co-authors from different organizations. We do not claim that this list is complete nor reflecting practices in every pharmaceutical company. However we believe it would be a good starting point. We have now expanded the description on the collection of liability motifs Table 1 and in the Methods section.

**2. The authors compare and contrast the distributions of the liabilities across the datasets and then subsequently present a flagging methodology for removing benign liabilities. It would make more sense to show the distributions of liabilities in each dataset after the flagging procedure is applied.**

Thank you for this comment, we have now extended Figure 3 with three panels indicating the proportion of each liability motif in all datasets after applying three individual flags separately.

**3. On line 239, the authors point to concerns related to CDR numbering schemes. Do the methods used to arrive at Table 1 use the same numbering scheme (IMGT)? Additionally, it would be useful to provide a reference for this, for example:
Dondelinger et al. Understanding the Significance and Implications of Antibody Numbering and Antigen-Binding Surface/Residue Definition, Front. Immunol., 9(2278)1-15, 2018**

Yes, we use the IMGT scheme throughout and the tests were to check in what numbers the liabilities can elude our method by not falling within this scheme. Thank you for the reference, we added it to the publication now as it gives substance to the statement on the differences between the numbering schemes.

**4. On line 294, the authors state "Aqueous solvents such as water are known to drive the chemical degradation of proteins." Neutral water by itself without co-solutes does not generally drive chemical degradation of proteins. Also, aqueous by definition refers exclusively to water and no other solvents. I believe the intention of the authors was to point to the fact that aqueous (water containing) solutions (not solvents) can lead to chemical degradation, so I think this should be rephrased to make it more clear.**

Thank you for the suggestion on aqueous solvents, however we must respectfully disagree with respect to water not driving chemical degradation. Based on Creighton’s Proteins: Molecular properties water indeed can be the driver of spontaneous chemical degradation in proteins. Indeed, Chapter 10 on page 463 in this book begins with these rather poetic sentences. “Although Proteins have many remarkable properties, they are not immortal. They eventually succumb to old age, brought on by gradual chemical modification in a variety of nonenzymatic chemical reactions.” Furthermore, Section 10.1 Chemical Aging on page 464 begins with the following sentences. “One factor that potentially limits the lifetime of a protein is its susceptibility to chemical change. The oxygen-rich, aqueous environments in which most proteins exist tend to produce a variety of chemical reactions in proteins.” We have now included the citation and included the aqueous solutions in the statements as suggested by the reviewer.

**5. The phrasing on line 235 is awkward, consider rephrasing to "which antibody region the liabilities in the germlines were in".**

Thank you for this suggestion. We have now rewritten this sentence.

Reviewer 2:  **1. The description of the germline flag does not indicate how the CDR3 region is handled. Is this region excluded when applying the flag since it is lacking in the germline sequence as it arises from recombination? If it is excluded and a significant fraction of liabilities arise from CDR3, perhaps the presence of the motif in "germline" sequences would be better handled through its frequency in the natural antibody space, similar to the therapeutic flag used in this study. Alternatively, a machine learning approach that accounts for the context may be more appropriate (see below).**

Thank you for this comment as the handling of H3 is a missing part from our publication. We do not include the D region because of difficulty in its identification and thus the liabilities are only those contained within the V and J genes. We have now reflected this in the publication in the methods section whilst describing the germline flag.

Thank you for the comment regarding the frequency in natural space, in fact this was the initial approach we have taken, off the back of popular language models such as ABlang (https://academic.oup.com/bioinformaticsadvances/article/2/1/vbac046/6609807) or our own statistics from AbDiver (https://academic.oup.com/bioinformatics/article/38/9/2628/6547051). Nevertheless we were dissuaded from this approach in favor of germlines. We were not convinced that any naturally observed mutation has a favorable liability profile - we would argue that it might be dependent on observed frequencies, with some motifs less likely because they might result in heterogeneous binding as a result of chemical modifications. Also, germlines are the common denominator in immunoglobulin frequency in an otherwise very differentiated and personal set of molecules- thus we argued that if something is allowed in germline, there is a strong biological foundation for that. Likewise, this is a point that we have left out of the paper but have now included it in the discussion section.

**2. Using the occurrence of a given motif at a given position within the natural and therapeutics antibody sequence space is predictive of whether a given motif will be a true chemical liability. However, the probability of occurrence for a given amino glosses over other factors, such as the identities of neighbouring amino acids in sequence and in structure. A machine learning method that could account for the context of a given position within the sequence, such as a sequence-based attention model, could potentially be more powerful in evaluating the presence of a given sequence within a larger sequence space, such as the natural antibody space.**

We agree with the reviewer. It would be ideal to train a model predicting liabilities based on wider sequence context. Nevertheless, the data that is available currently is tens of data points both for training and testing. We also argue that generating more data would not only need to focus on specific modifications but also parametrize the different conditions. We have now added such an argument to the discussion.

**3. The manuscript would benefit from more detailed descriptions of the figures and tables, and a more detailed description of the flags in the core of the paper in addition to the methods section.**

We have now extended the descriptions for tables and figures throughout the manuscript. We also extended the descriptions of columns in Antibody Liability Reference (Table 1) and expanded Figure 4 to have lambda and kappa chains.
